# Reported definitions of intraoperative hypotension in adults undergoing non-cardiac surgery under general anaesthesia: a review

**DOI:** 10.1186/s12871-022-01605-9

**Published:** 2022-03-11

**Authors:** Laurence Weinberg, Stephanie Ying Li, Maleck Louis, Jadon Karp, Nadia Poci, Bradly Samuel Carp, Lachlan Fraser Miles, Patrick Tully, Robert Hahn, Dharshi Karalapillai, Dong-Kyu Lee

**Affiliations:** 1 Department of Anaesthesia, Austin Hospital, Austin Health, 145 Studley Road, Heidelberg, Melbourne, VIC 3084 Australia; 2 Department of Critical Care, The University of Melbourne, Melbourne, Australia; 3 Department of Surgery, Austin Health, The University of Melbourne, Melbourne, Australia; 4 Karolinska Institute at Danderyd’s Hospital (KIDS), Stockholm, Sweden; 5 Department of Research, Södertälje Hospital, Södertälje, Sweden; 6 Department of Anesthesiology and Pain Medicine, Dongguk University Ilsan Hospital, Goyang, Republic of Korea

**Keywords:** Hypotension, Anesthesia, Blood pressure, Surgery

## Abstract

**Background:**

Intraoperative hypotension (IOH) during non-cardiac surgery is common and associated with major adverse kidney, neurological and cardiac events and even death. Given that IOH is a modifiable risk factor for the mitigation of postoperative complications, it is imperative to generate a precise definition for IOH to facilitate strategies for avoiding or treating its occurrence. Moreover, a universal and consensus definition of IOH may also facilitate the application of novel and emerging therapeutic interventions in treating IOH. We conducted a review to systematically record the reported definitions of intraoperative hypotension in adults undergoing non-cardiac surgery under general anaesthesia.

**Methods:**

In accordance with Cochrane guidelines, we searched three online databases (OVID [Medline], Embase and Cochrane Library) for all studies published from 1 January 2000 to 6 September 2020. We evaluated the number of studies that reported the absolute or relative threshold values for defining blood pressure. Secondary aims included evaluation of the threshold values for defining IOH, the methodology for accounting for the severity of hypotension, whether the type of surgical procedure influenced the definition of IOH, and whether a study whose definition of IOH aligned with the Perioperative Quality Initiative-3 workgroup (POQI) consensus statement for defining was more likely to be associated with determining an adverse postoperative outcome.

**Results:**

A total of 318 studies were included in the final qualitative synthesis. Most studies (*n* = 249; 78.3%) used an absolute threshold to define hypotension; 150 (60.5%) reported SBP, 117 (47.2%) reported MAP, and 12 (4.8%) reported diastolic blood pressure (DBP). 126 (39.6%) used a relative threshold to define hypotension. Of the included studies, 153 (48.1%) did not include any duration variable in their definition of hypotension. Among the selected 318 studies 148 (46.5%) studies defined IOH according to the POQI statement. When studies used a “relative blood pressure change” to define IOH, there was a weaker association in detecting adverse postoperative outcomes compared to studies who reported “absolute blood pressure change” (χ^2^(2) = 10.508, *P* = 0.005, Cramér’s *V* = 0.182). When studies used the POQI statement definition of hypotension or defined IOH by values higher than the POQI statement definition there were statistical differences observed between IOH and adverse postoperative outcomes (χ^2^(1) = 6.581, *P* = 0.037, Cramér’s *V* = 0.144). When both the duration of IOH or the numbers of hypotensive epochs were evaluated, we observed a significantly stronger relationship between the definition of IOH use the development of adverse postoperative outcomes. (χ^2^(1) = 4.860, *P* = 0.027, Cramér’s *V* = 0.124).

**Conclusions:**

Most studies defined IOH by absolute or relative changes from baseline values. There are substantial inconsistencies in how IOH was reported. Further, definitions differed across different surgical specialities. Our findings further suggest that IOH should be defined using the absolute values stated in the POQI statement i.e., MAP < 60–70 mmHg or SBP < 100 mmHg. Finally, the number of hypotensive epochs or time-weighted duration of IOH should also be reported.

**Supplementary Information:**

The online version contains supplementary material available at 10.1186/s12871-022-01605-9.

## Background

### Rationale

Intraoperative hypotension (IOH) during non-cardiac surgery is common and associated with major adverse kidney, neurological and cardiac events and even death [1, 2]. Given that IOH is a modifiable risk factor for the mitigation of postoperative complications, it is imperative to generate a precise definition for IOH to facilitate strategies for avoiding or treating its occurrence. Moreover, a universal and consensus definition of IOH may also facilitate the application of novel and emerging therapeutic interventions in treating IOH. To date, a consensus definition of IOH remains elusive.

No recent studies have examined reported definitions of IOH. However, Bijcker et al. reported 140 different definitions of IOH provided in over 100 studies over the period January 2000 to April 2006 [3]. Further, the methods of presenting IOH also differ significantly, from counting the number of hypotensive episodes or measuring the duration or severity of hypotension, to using a combination of both [4].

### Objectives

The primary aim of this review was to describe the definitions of IOH used in the contemporary literature for adult patients undergoing non-cardiac surgery under general anaesthesia.

## Methods

### Protocol and registration

This review was conducted using similar methodology to the Cochrane guidelines [5]. We have reported our findings using the guidance of the Preferred Reporting Items for Systematic Reviews and Meta-Analyses (PRISMA) statement [6]. The protocol was prospectively registered with the international prospective register of reviews (PROSPERO ID: CRD42020204661; registered 17 September 2020; available from: https://www.crd.york.ac.uk/prospero/display_record.php?RecordID=204661).

### Search strategy

Three online databases (OVID [Medline], Embase and Cochrane Library) were searched on 6 September 2020, based on their applicability to the fields of health and medicine. A search strategy was constructed combining free-text terms and Medical Subject Headings (MeSH) (see Additional file 1: Table 1. Search strategy). All studies published from 1 January 2000 to 6 September 2020 were included, and searches were conducted without language limiters.

### Search terms

We used keyword search terms that focused on ‘surgery and hypotension’ and the various permutations of how IOH can be described—that is, absolute or relative changes in systolic, diastole or mean arterial pressure, or any combination thereof. We also used the MeSH terms to screen for all intraoperative complications from surgery and general anaesthesia. After a preliminary search, the specific terms ‘mean arterial pressure’ (MAP) and ‘MAP’ were included in our search strategy, as studies frequently reported indications for anti-hypotensive treatment using MAP thresholds without explicitly stating ‘hypotension’. Additionally, the reference lists of all identified studies were searched for additional studies.

### Eligibility criteria

We included all studies that investigated adult patients undergoing non-cardiac surgery under general anaesthesia that reported a definition for IOH. These included randomised and non-randomised controlled trials, comparative observational studies, case–control studies and cohort studies. Conference abstracts were also included when all inclusion criteria were met. Studies that investigated multiple forms of anaesthesia, where one of those forms included general anaesthesia, were also included. Studies of adult patients (18 years or older) were targeted; however, studies including a combination of paediatric and adult populations, or adults undergoing cardiac and non-cardiac surgery, were included to extract data from the adult non-cardiac population cohort.

We excluded editorials, opinions and idea-based articles. We also excluded systematic reviews and meta-analyses to avoid duplication of data. Case reports and case series of fewer than 10 patients were excluded because reported definitions were not deemed generalisable. Papers were excluded if there was insufficient information in the English-language abstract or if they were not published in English. Finally, studies were also excluded if the anaesthetic technique involved deliberate induction of hypotension.

### Primary and secondary aims

The primary aim was to evaluate the number of studies that reported the absolute or relative threshold values for defining blood pressure. The secondary aims included evaluation of i) the threshold values for defining IOH; ii) the methodology for accounting for the severity of hypotension (e.g., duration of low blood pressure, number of hypotensive events, nadir blood pressure or total duration of hypotension); iii) whether the type of surgical procedure influenced the definition of IOH; and iv) whether a study whose definition of IOH aligned with the Perioperative Quality Initiative-3 workgroup (POQI) consensus statement for defining intraoperative hypotension (i.e. a MAP < 20% of baseline or a MAP<70mmhg) [7] was more likely to be associated with determining an adverse postoperative outcome.

### Data collection process and data items

All studies were imported into a web-based software platform (Covidence®, Veritas Health Innovation, Melbourne, Australia), which supported citation screening, full-text review and removal of duplicated data. Two researchers (SL, ML) independently screened the retrieved studies based on their title and abstract against the pre-specified inclusion and exclusion criteria. Any conflict was resolved by a third researcher (LW). Full-text articles were retrieved for those studies selected for inclusion. These studies were evaluated again against the inclusion and exclusion criteria. The data from studies coded ‘include’ were extracted into a formatted table; five researchers (SL, JK, NP, PT, LW) participated in the data extraction. Data extraction was piloted on a subset of the search results to optimise the data extraction process.

### Definitions of hypotension and synthesis of results

Definitions of hypotension were predominantly based on either systolic blood pressure (SBP), MAP or a combination of the two. Studies reporting definitions of hypotension exhibited four points of contention: i) choice of an absolute threshold value for defining hypotension, ii) choice of a value for defining hypotension relative to a change from a baseline value, iii) definition of baseline blood pressure for reporting relative threshold values and iv) methodology for characterising severity. The results are reported in the form of a narrative synthesis.

### Definition of baseline blood pressure measurement

Baseline blood pressure measurements were defined as either the initial blood pressure in the operating room as a baseline value, or the blood pressure value measured at the preoperative visit within 30 days before surgery.

### Risk of bias across studies

Given that the present study only reports the definition of IOH used in any given published study, and our review is not a meta-analysis, a risk of bias was not assessed. In addition, we expected a high degree of heterogeneity between studies as we planned to include studies from multiple procedure types across all adult surgical specialties. As such, no formal assessment of bias within studies was planned or expected to be possible.

### Statistical analysis

Statistical analysis was performed using IBM SPSS Statistics for Windows, version 23 (IBM Corp., 2015, Armonk, NY, USA). Data was presented using descriptive statistics and presented a number (proportion). To evaluate whether each study’s reported definition of IOH was aligned with POQI consensus statement for defining hypotension (i.e., a MAP < 20% of baseline or a MAP <70mmhg), and if such definitions were more likely to be associated with determining adverse postoperative outcomes, we further categorised each study’s definition of IOH into three predictor variables namely, i) the type of IOH, ii) whether the definition of IOH was the same or different to the POQI consensus statement for defining IOH, and iii) whether studies reported the number of epochs and the duration of hypotension.

The type of IOH was categorised into 3 variables: absolute, relative, or a combination of the two. The definition of IOH was categorised as “the same” if it was the identical to the POQI consensus definition for IOH, “higher” when the defined blood pressure threshold value was higher than the POQI statement definition for IOH, or “lower” when the defined blood pressure threshold value was lower than the statement. If the study evaluated the number of hypotensive epochs or the duration of each hypotensive event, we further categorised this by “considered” or “not considered”. Finally, if the study’s predefined definition of IOH was associated with an adverse patient outcome, e.g., AKI, mortality etc., we categorised this as “validated” or “not-validated”.

The relationship between postoperative adverse outcomes and the predefined definition of IOH was evaluated using chi-squared analysis and binary logistic regression. The three predictor variables were tested a priori to verify if there was any possible multicollinearity with the correlation analysis i.e., if each independent variable is computed from other variables in the data set, or if two independent variables provide similar and repetitive results. The estimated model was evaluated with Hosmer-Lemeshow statistics for the goodness-of-fit, and estimated coefficients were evaluated using Wald statistic and corresponding *P* values. Residuals were evaluated with plots of the standardised residuals and Cook’s influence statistics. A two-sided *P* **<**value lower than 0.05 was considered statistically significance. Inferred statistical results were presented with corresponding effect sizes.

## Results

### Study selection

**Fig. 1:**
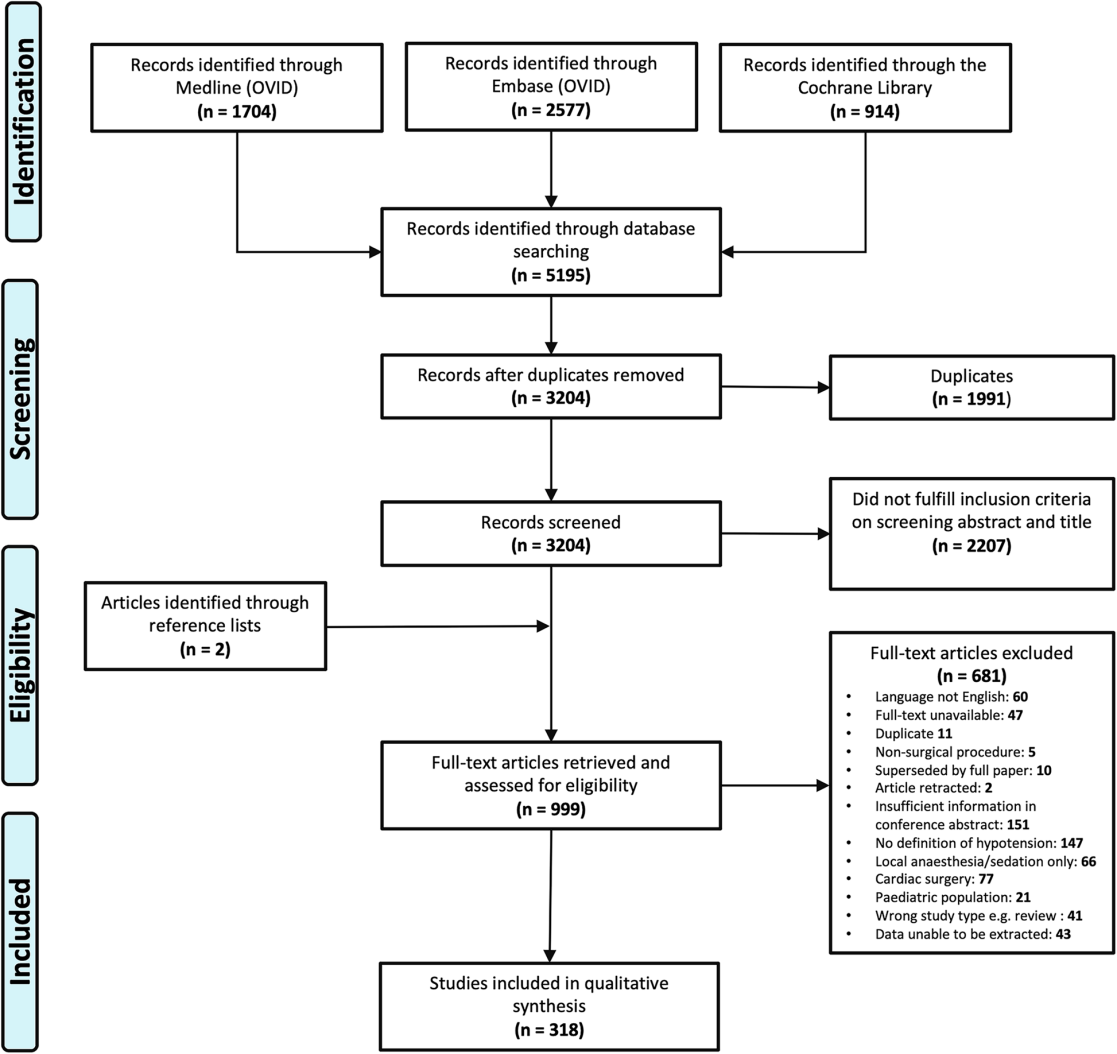
Study flow diagram

Initial searching yielded 5195 studies; 1704 were identified through Medline, 2577 were identified through Embase, and 914 were identified through Cochrane Library. The search histories for all databases are presented in the flow diagram (see Fig. 1). A total of 318 studies were included in the final qualitative synthesis. The full data sheet for all included papers is available as an additional file (see Additional file 2: Excel Sheet. Full dataset).

### Study characteristics

The included studies exhibited significant heterogeneity. Studies originated from 42 countries, with over half originating from one of five countries: USA, China, Japan, South Korea and the Netherlands. Most studies were presented in journal papers. The characteristics and primary outcomes of all included studies are presented in Table 1.

**Table 1:** Summary of studies reporting definitions of intraoperative hypotension

Study characteristic		No of studies (total = 318)
Country	United States	88 (27.7%)
	China	30 (9.4%)
	Japan	24 (7.5%)
	Korea South	21 (6.6%)
	Netherlands	16 (5%)
	France	15 (4.7%)
	United Kingdom	14 (4.4%)
	India	11 (3.5%)
	Canada	10 (3.1%)
	Germany	10 (3.1%)
	others	79 (24.8%)
Type of publication	Journal paper	292 (91.8%)
	Conference abstract	26 (8.2%)
Study design	Retrospective cohort	138 (43.4%)
	Prospective cohort	82 (25.8%)
	Prospective RCT	73 (23%)
	Retrospective case–control	13 (4.1%)
	Retrospective audit	4 (1.3%)
	Prospective audit	3 (0.9%)
	Prospective case-cohort	2 (0.6%)
	Not specified	3 (0.9%)
Study setting	Single centre	267 (84%)
	Multicentre	37 (11.6%)
	Not specified	14 (4.4%)
Study sample size	Minimum	10 participants
	Maximum	147,573 participants
Monitoring technique (Some studies included more than one technique)	Invasive, arterial line	110 (30.1%)
	Non-invasive, intermittent	93 (25.4%)
	Non-invasive, continuous	12 (3.3%)
	Not specified	151 (41.3%)

### Absolute thresholds for intraoperative hypotension

**Fig. 2:**
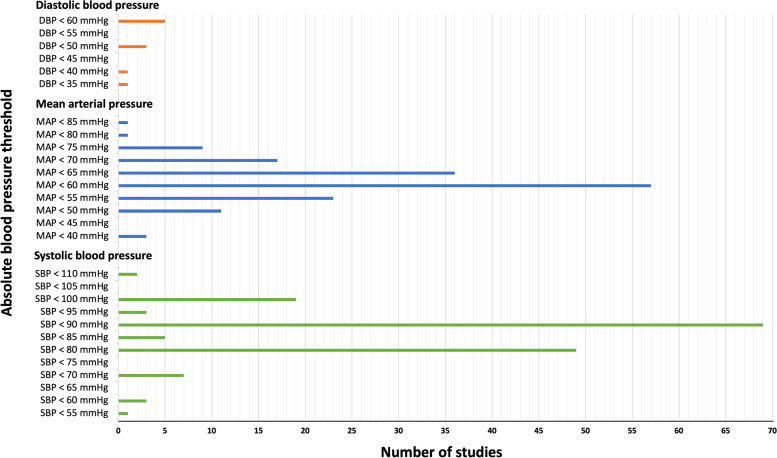
Absolute numerical thresholds for intraoperative hypotension in the included studies. Several articles used more than one definition

Most studies (*n* = 249; 78.3%) used an absolute threshold to define hypotension; 150 (60.5%) reported SBP, 117 (47.2%) reported MAP, and 12 (4.8%) reported diastolic blood pressure (DBP). The majority of studies (221, 89.1%) reported a single component of the measured blood pressure (i.e., either MAP, SPB or DBP) as a threshold. Twenty-five studies (10.1%) reported two blood pressures components, and two studies (0.8%) reported three. Reported SBP thresholds ranged from 55 mmHg [8] to 110 mmHg [9, 10]. MAP thresholds ranged from 40 mmHg [11–14] to 85 mmHg [15]. DBP thresholds included 35 mmHg, 40 mmHg [16], 50 mmHg [17–19] and 60 mmHg [20–24]. The most frequently used definitions were an SBP below 90 mmHg (*n* = 69, 43.4% of reported SBP), a MAP below 60 mmHg (*n* = 57, 35.4% of reported MAP). The incidence of different absolute numerical thresholds for hypotension is presented in Fig. 2. The definitions of absolute thresholds for IOH across different surgeries are summarised in Table 2.

**Table 2:** Defining intraoperative hypotension across different surgical specialties

Type of surgery (alpahbetics order)	Number (%) of total studies (***n*** = 318)	Blood pressure	Absolute blood pressure value in mmHg below which hypotension was defined		Relative % change from baseline blood pressure value to define hypotension	
			Reported number^**a**^	Commonly reported blood pressure value (Number of studies)^b^	Reported number^**a**^	Commonly reported blood pressure value (Number of studies) ^b^
Abdominal surgery	56 (17.6%)	Systolic	40	90 (15), 80 (14)	9	20 (4), 30 (3)
		Mean	18	60 (9), 65 (5)	5	30 (2), 15 (1), 20 (1), 50 (1)
		Diastolic	3	60 (3)	0	–
Ear, nose and throat (ENT) surgery and head, neck, maxillary-facial surgery	11 (3.5%)	Systolic	3	70 (1), 80 (1), 90 (1), 100 (1)	6	30 (3), 20 (2)
		Mean	6	60 (4), 50 (1), 55 (1)	5	30 (3), 20 (1), 40 (1)
		Diastolic	2	40 (1), 50 (1)	2	30 (2)
General surgery	60 (18.9%)	Systolic	27	90 (12), 70 (3)	24	20 (9), 30 (6)
		Mean	43	65 (12), 60 (11)	21	20 (6), 30 (5)
		Diastolic	0	–	1	20 (1)
Gynaecological surgery	11 (3.5%)	Systolic	5	80 (2), 90 (2)	4	20 (3), 30 (1)
		Mean	8	60 (3), 65 (2), 75 (2)	1	20 (1)
		Diastolic	0	–	1	20 (1)
Other surgeries (plastic, trauma, ophthalmology)	10 (3.1%)	Systolic	7	90 (5), 80 (1), 110 (1)	1	20 (1)
		Mean	4	55 (2), 60 (1), 65 (1)	2	20 (1), 30 (1)
		Diastolic	0	–	0	–
Orthopaedic surgery	46 (14.5%)	Systolic	25	90 (11), 80 (9)	19	20 (10), 30 (4)
		Mean	23	60 (9), 55 (7)	13	20 (5), 30 (4)
		Diastolic	2	60 (1), 35 (1)	3	30 (1), 40 (1), 50 (1)
Spinal surgery	14 (4.4%)	Systolic	2	80 (1), 85 (1)	1	20 (1)
		Mean	11	55 (3), 60 (2), 65 (2), 70 (2)	5	20 (2), 25 (1), 30 (1), 35 (1), 40 (1)
		Diastolic	0	–	0	–
Thoracic surgery	12 (3.8%)	Systolic	4	90 (2), 80 (1), 100 (1)	3	30 (2), 20 (1)
		Mean	10	60 (4), 55 (3)	5	20 (2), 25 (1)
		Diastolic	0	–	0	–
Transplantation	17 (5.3%)	Systolic	7	90 (3), 70 (1), 80 (1), 95 (1), 100 (1)	4	20 (1), 30 (1), 33 (1), 50 (1)
		Mean	8	60 (3), 70 (2)	1	20 (1)
		Diastolic	1	50 (1)	1	20 (1)
Urological surgery	9 (2.8%)	Systolic	3	90 (2), 80 (1)	4	20 (2), 30 (1), 40 (1)
		Mean	7	65 (3), 60 (2)	3	20 (2), 30 (1)
		Diastolic	0	–	0	–
Vascular surgery	41 (12.9%)	Systolic	25	80 (8), 90 (8), 100 (6)	5	20 (3), 30 (2)
		Mean	15	60 (6), 65 (3), 70 (3)	8	30 (3), 20 (2), 40 (2)
		Diastolic	0	–	0	–

^a^: Number of corresponding blood pressure components used to define IOH ^b^: Frequently used values and reported number of studies. Some studies have multiple threshold definitions of intraoperative hypotension)

### Relative thresholds for intraoperative hypotension

**Fig. 3:**
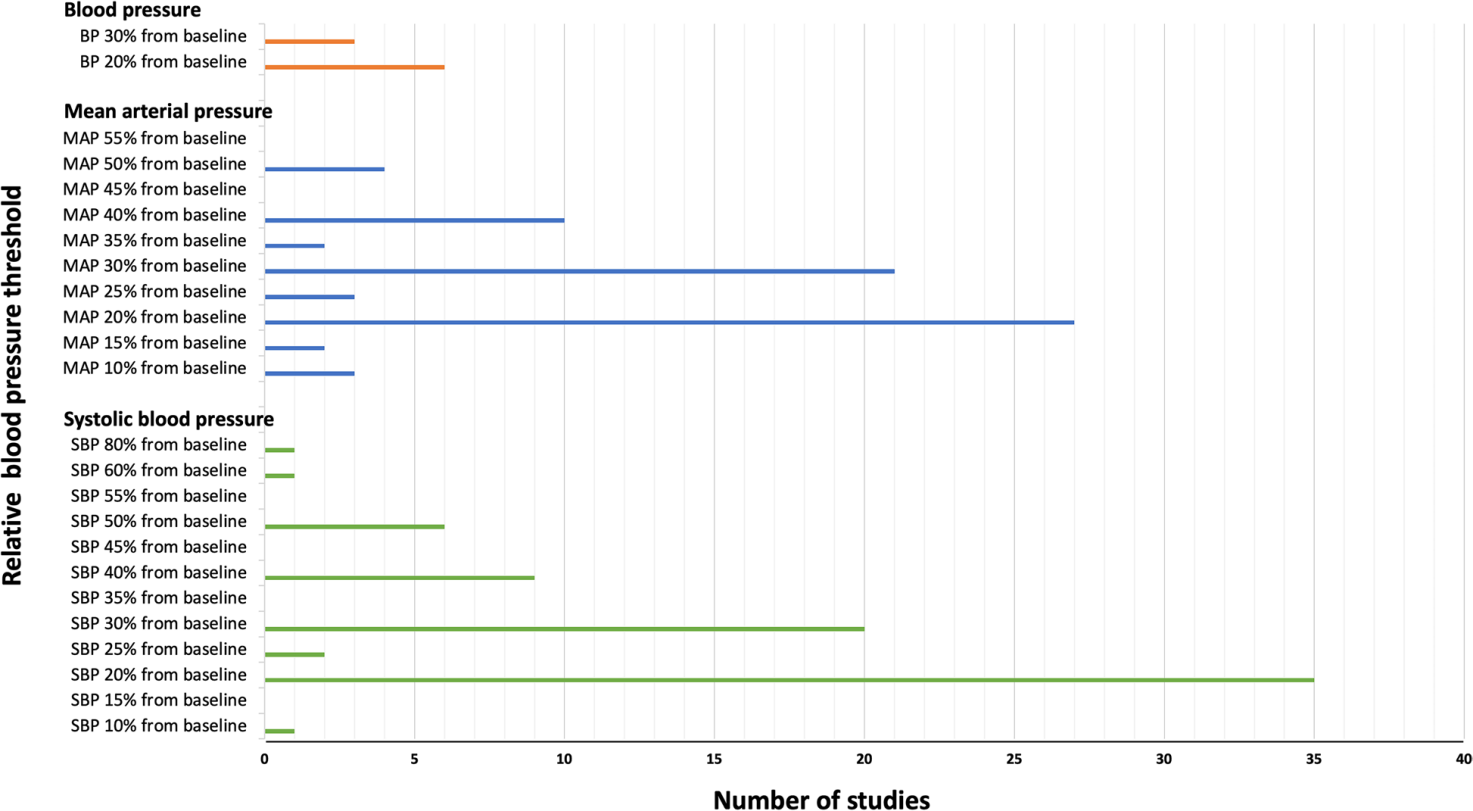
Relative thresholds for intraoperative hypotension in the included studies. Several articles used more than one definition

Of the included studies, 126 (39.6%) used a relative threshold to define hypotension. Most studies used a percentage threshold, with only five using absolute decreases from the baseline [18, 25–28]. Three studies used a combination of a relative decrease and an absolute threshold [29–31]. SBP thresholds varied from 10% [11] to 60% [14] decreases from baseline, and MAP thresholds varied from 10% [11, 32, 33] to 60% [14] decreases from baseline. The most frequently used definitions were 20% decreases from baseline SBP (*n* = 35) or MAP (*n* = 27). The incidence of different relative thresholds for hypotension is presented in Fig. 3. Of the 126 (39.6%) studies that used a relative threshold, 87 (69.0%) presented a baseline time reference point, the most common of which was the pre-induction pressure or pre-surgical manipulation. However, other reported definitions included blood pressure measurements taken in pre-admission clinics or on the wards. The definitions of relative thresholds for IOH across different surgeries are summarised in Table 2.

### Other definitions of hypotension

The search strategy identified 10 articles that defined hypotension as a blood pressure requiring a therapy (e.g., fluids or vasopressors) to be administered by the attending anaesthesiologist [25, 34–42].

### Methodology for calculation of intraoperative hypotension severity

**Fig. 4:**
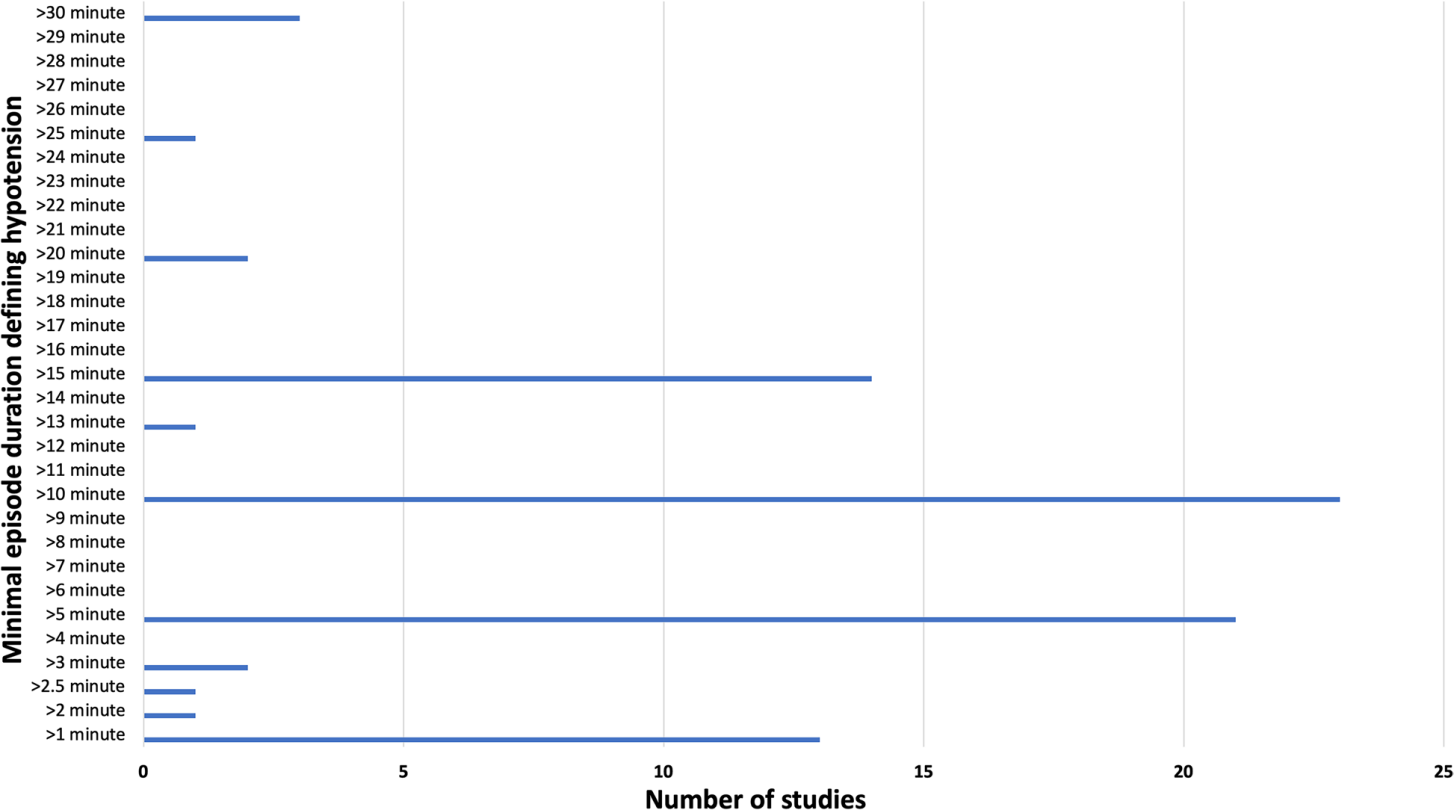
Minimal episode duration included in the definition of hypotension in the included studies

Of the included studies, 153 (48.1%) did not include any duration variable in their definition of hypotension. Eighty-five definitions incorporated a minimal episode duration in their definition; however, these measurements ranged from a duration of 1 min to 30 min of hypotension, with the most used duration being 10 min (*n* = 23). The incidence of different episode lengths for clinically significant hypotension is presented in Fig. 4. Other methodologies included measurement of the number of episodes of hypotension (*n* = 28), the nadir blood pressure (*n* = 9), total duration of hypotension or a combination of duration and severity (*n* = 135), expressed as a time-weighted sum or an area under the curve (*n* = 19).

### The relationship between the various definitions of intraoperative hypotension reported in the literature and their association with the development of adverse postoperative outcomes

One hundred and sixteen studies (36.5%) reported a statistically significant relationship between IOH and adverse postoperative outcomes. Among the selected 318 studies, 192 (60.4%) used the absolute blood pressure values, 69 (21.7%) used relative blood pressure changes, and 57 (17.9%) used both the absolute and relative blood pressure changes (Table 3). When studies used a “relative blood pressure change” to define IOH, there was a weaker association in detecting adverse postoperative outcomes compared to studies that reported the “absolute blood pressure change” (*χ*^2^(2) = 10.508, *P* = 0.005, Cramér’s *V* = 0.182)*.* Among selected 318 studies, 148 (46.5%) studies defined IOH according to the POQI statement [7] i.e., a MAP < 60–70 mmHg, a SBP < 100 mmHg, or a 20% change from the baseline blood pressure measurements), 11 (3.5%) and 158 (49.7%) studies used higher or lower blood pressure thresholds, respectively. When studies used defined IOH by values higher than the POQI statement definition, there was a stronger association in detecting adverse postoperative outcomes compared to concordant or lower thresholds definition (*χ*^2^(1) = 6.581, *P* = 0.037, Cramér’s *V* = 0.144).

**Table 3:** The relationship between the reported definitions of intraoperative hypotension and their association with adverse postoperative outcomes

Categorised IOH definitions		The development of an adverse postoperative outcomes	
		Not associated	Associated
Type of IOH definitions (*n* = 318)*	Absolute	111 (57.8%)	81 (42.2%)
	Relative	55 (79.7%)	14 (20.3%)
	Combined	36 (63.2%)	21 (36.8%)
Concordant with the POQI statement for defining IOH (*n* = 317) *,^a^	Concordant	97 (65.5%)	51 (34.5%)
	Lower	102 (64.6%)	56 (35.4%)
	Higher	3 (27.3%)	8 (72.7%)
The number of epochs and the duration of hypotension (n = 318)*	No	89 (57.4%)	66 (42.6%)
	Yes	113 (69.3%)	50 (30.7%)

Data are presented as a number (percentile). * *P* < 0.05 with the chi-squared test, ^a^ 1 studies was excluded due to study-specific threshold. IOH: Intraoperative hypotension, POQI statement: Perioperative Quality Initiative-3 workgroup statement [7]

One hundred sixty-three (51.3%) studies reported hypotension “duration” or “number of hypotensive episodes” to define IOH. Studies that did not report “duration” or “number of hypotensive episodes” in their IOH definition had a stronger association between IOH and the development of adverse postoperative outcomes (χ^2^(1) = 4.860, *P* = 0.027, Cramér’s *V* = 0.124). The estimated ORs showed that using “relative changes” only to define IOH definition and excluding both the “number of epochs” and the “duration of hypotension” were less likely to predict poor adverse postoperative outcomes (Table 4).

**Table 4:** Definitions of intraoperative hypotension reported in the literature and the prediction of development of adverse postoperative outcomes

Predictors		ß (SE)	Odds ratio (95% confidence interval)	***P***-value
Type of IOH	Absolute	(Reference)		
	Relative	−1.07 (0.35)	0.34 (0.17–0.68)	0.002*
	Combined	−0.10 (0.32)	0.91 (0.49–1.69)	0.752
Agreeable thresholds with POQI statement	Lower	(Reference)		
	Agreeable	−1.72 (0.72)	0.18 (0.04–0.73)	0.016*
	Higher	−1.60 (0.72)	0.20 (0.05–0.82)	0.025*
No consideration for the number of epochs and the duration of hypotension		−0.71 (0.20)	0.49 (0.33–0.73)	< 0.001*

*: *P* < 0.05 by binary logistic regression. -2LL = 393.4, Cox & Snell R^2^ = 0.067, Hosmer and Lemeshow test *P* = 0.766. *IOH* Intraoperative hypotension, *POQI statement* Perioperative Quality Initiative-3 workgroup statement [7]. Absolute: IOH defined by an absolute blood pressure value, Relative: IOH defined by the relative changes from baseline blood pressure, Lower: IOH threshold having lower values compared to POQI statement, Agreeable: IOH thresholds having concordant values to POQI statement, Higher: IOH thresholds having higher values compared to the POQI statement

## Discussion

### Key findings

A review was conducted of 318 studies assessing the definitions of hypotension used in adult patients undergoing non-cardiac surgery under general anaesthesia. We found substantial inconsistencies in ranges of the reported definitions of IOH. The most frequently reported definitions of IOH were an SBP < 90 mmHg, MAP < 60 mmHg and a 20% decrease from the baseline measurement of either MAP or SBP. Less than 50% of studies conformed to the POQI statement recommendations for defining IOH [7]. A higher or lower IOH threshold compared to the definition used in the POQI statement did not increase the accuracy in detecting postoperative complications. Therefore, our findings support the “absolute value definition” used in the POQI statement to define IOH. In adidtion, the numbers of hypotensive epochs, and the absolute duration or time-weighted duration of IOH should also be reported.

Our review highlights significant limitations in the defining and reporting of IOH. First, hypotension was not defined in many studies that reported it as an outcome. Second, the definitions of IOH across the studies varied with little consensus. Third, because the reported definitions varied, direct comparison of the incidence of IOH and the adverse sequelae of IOH between studies was not possible.

To mitigate the associations of IOH and adverse outcomes in non-cardiac surgery, the Perioperative Quality Initiative reported a consensus that intraoperative MAPs < 60–70 mmHg are associated with myocardial injury, acute kidney injury, and death. Systolic arterial pressures < 100 mmHg are associated with myocardial injury and death. Therefore, organ injury appears to be a function of both hypotension severity and hypotension duration [7]. As evidenced by our findings, there was significant heterogeneity in patient and procedural risk factors across the studies that reported IOH. Significant differences between the patient populations and surgical procedures selected for review limited subgroup analysis. We found that some studies targeted highly specific patient populations, such as the very elderly, [43] while others targeted specific surgical techniques [44]. Moreover, others only examined hypotension thresholds for syndromes [45]. Additionally, using the initial blood pressure in the operating room as a baseline value may be inaccurate and misleading due to the high incidence of white-coat induced hypertension, resulting in an erroneously high blood pressures recording.

While this review focused primarily on determining the most common reported definition of hypotension, Bijker et al. [3] have suggested that threshold values and duration of IOH may differ across different postoperative outcomes. Our study supports the findings of a recent review by Wesselink et al., [46] who reported that making quantitative associations between intraoperative hypotensive events and postoperative outcomes was hampered by the heterogeneity of definitions used. Future work exploring the degree and duration of hypotension to be avoided for averting specific postoperative adverse events may reveal significant differences in blood pressure targets. Prospective interventional studies showing causative associations between IOH and postoperative outcomes are necessary to support the existing evidence base and improve clinical practice.

Our study has several strengths and limitations. Many studies reporting on IOH were excluded because they failed to provide an explicit definition. Further, most (73%) of the included studies in this review were published before the 2019 POQI statement [7], which was published in 2019. Nevertheless, the present review is the most extensive study to date reporting on the definitions of IOH. Accordingly, the high number of articles identified allows an accurate overview of the reported definitions of IOH across different countries, surgeries and adult populations. The most common type of study was a retrospective cohort study, and most studies were observational. Therefore, we are unable to draw any associations between the occurrence of IOH, the types of surgery or patient factor and adverse outcomes. The findings of this study are not generalisable to paediatric populations, adult patients undergoing cardiac surgery or studies where the anaesthetic technique involves deliberate induction of hypotension.

Our review has important clinical implications. First, our findings support a more standardised approach for defining IOH. Studies should also follow a detailed and consistent methodology when reporting IOH, using reported consensus definitions [7]. Finally, as a minimum, we advocate that both SBP and MAP values should be used when reporting IOH. The number of hypotensive epochs that occur and the total duration of time that patients spend below the predefined hypotensive thresholds should also be reported. This will allow comparisons of the adverse sequelae of hypotension across different patient and surgical cohorts.

## Conclusion

We conclude that most studies defined IOH by absolute or relative changes from baseline values; however, this review identified substantial inconsistencies in how IOH was reported. Further, definitions differed across different surgical specialities. Despite this variability, we found the most frequently reported definitions of IOH were an SBP < 90 mmHg, a MAP < 60 mmHg or a 20% decrease from the baseline measurement of either MAP or SBP. Our findings further suggest that IOH should be defined using the absolute values stated in the POQI statement [7] i.e., MAP < 60–70 mmHg or SBP < 100 mmHg. Finally, the number of hypotensive epochs or time-weighted duration of IOH should also be reported. Future studies incorporating such definitions should be designed to further evaluate individualised blood pressure targets for both patient and surgery factors, together with organ-specific blood pressure outcome metrics.

## Supplementary Information

**Additional file 1: Supplementary Table.** Search strategy used for the review and number of results.

**Additional file 2.** Full dataset.

## Data Availability

The datasets generated and analysed during the study are not publicly available due to individual privacy concerns but are available from the corresponding author on reasonable request.

## Abbreviations

DBP: Diastolic blood pressure
IOH: Intraoperative hypotension
MAP: Mean arterial pressure
POQI: Perioperative Quality Initiative-3 workgroup
SBP: Systolic blood pressure
