# Supplementary material for: Reported definitions of intraoperative hypotension in adults undergoing non-cardiac surgery under general anaesthesia: a review
Source: BMC Anesthesiol. 2022 Mar 11;22:69. doi: 10.1186/s12871-022-01605-9 (PMC8915500; doi:10.1186/s12871-022-01605-9)
Supplement: Supplementary file 1 — Additional file 1: Supplementary Table. Search strategy used for the review and number of results. [file 12871_2022_1605_MOESM1_ESM.docx]

**Supplementary Table:** Search strategy used for the review and number of results

|  | **Cochrane Library** | **Results** |
| --- | --- | --- |
| 1 | hypotens* NEAR/3 (intraoperative OR intra-operative OR perioperative OR peri-operative OR peroperative OR per-operative OR surg*) | 847 |
| 2 | ((low OR decreased OR reduced) NEXT (blood NEXT pressure*)) NEAR/3 (intraoperative OR intra-operative OR perioperative OR peri-operative OR peroperative OR per-operative OR surg*) | 7 |
| 3 | ((low OR decreased OR reduced) NEXT (arterial NEXT pressure*)) NEAR/3 (intraoperative OR intra-operative OR perioperative OR peri-operative OR peroperative OR per-operative OR surg*) | 1 |
| 4 | “arterial hypotension” AND (intraoperative OR intra-operative OR perioperative OR peri-operative OR peroperative OR per-operative OR surg*) | 125 |
| 5 | (intraoperative OR intra-operative OR perioperative OR peri-operative OR peroperative OR per-operative OR surg*) NEXT (“mean arterial pressure” OR “MAP”) |  |
| 6 | OR (1 - 5) (limit to studies conducted from 2000 onwards) | 914 |

|  | **Medline (OVID)** | **Results** |
| --- | --- | --- |
| 1 | hypotens* | 82207 |
| 2 | (low or decreased or reduced) adj1 blood pressure* | 8395 |
| 3 | (low or decreased or reduced) adj1 arterial pressure* | 1531 |
| 4 | 1 or 2 or 3 | 90440 |
| 5 | intraoperative or intra-operative or perioperative or peri-operative or  peroperative or per-operative or surg* | 3229434 |
| 6 | (intraoperative or intra-operative or perioperative or peri-operative or peroperative or per-operative or surg*) adj3 (hypotens* or ((low or decreased or reduced) adj1 blood pressure*) or ((low or decreased or reduced) adj1  arterial pressure*)) | 1918 |
| 7 | arterial hypotension | 2021 |
| 8 | 5 and 7 | 524 |
| 9 | mean arterial pressure or MAP | 228497 |
| 10 | (intraoperative or intra-operative or perioperative or peri-operative or  peroperative or per-operative or surg*) adj1 (mean arterial pressure or MAP) | 199 |
| 11 | exp Surgical Procedures, Operative/ae, co, ph [Adverse Effects,  Complications, Physiology] | 451482 |
| 12 | Hypotension/ | 22078 |
| 13 | Anesthesia, General/ | 40017 |
| 14 | Intraoperative Complications/ | 32151 |
| 15 | 11 and 12 and 13 and 14 | 24 |
| 16 | 6 or 8 or 10 or 15 | 2574 |
| 17 | limit 16 to yr=”2000 - Current” | 1704 |

|  | **Embase (OVID)** | **Results** |
| --- | --- | --- |
| 1 | hypotens* | 196033 |
| 2 | (low or decreased or reduced) adj1 blood pressure* | 11819 |
| 3 | (low or decreased or reduced) adj1 arterial pressure* | 1953 |
| 4 | 1 or 2 or 3 | 206104 |
| 5 | intraoperative or intra-operative or perioperative or peri-operative or  peroperative or per-operative or surg* | 4579936 |
| 6 | (intraoperative or intra-operative or perioperative or peri-operative or peroperative or per-operative or surg*) adj3 (hypotens* or ((low or decreased or reduced) adj1 blood pressure*) or ((low or decreased or reduced) adj1  arterial pressure*)) | 2686 |
| 7 | arterial hypotension | 3168 |
| 8 | 5 and 7 | 801 |
| 9 | mean arterial pressure or MAP | 277550 |
| 10 | (intraoperative or intra-operative or perioperative or peri-operative or  peroperative or per-operative or surg*) adj1 (mean arterial pressure or MAP) | 272 |
| 11 | exp Surgery/ | 5380593 |
| 12 | Hypotension/ | 127837 |
| 13 | Anesthesia, General/ | 80322 |
| 14 | Intraoperative Complications/ | 17046 |
| 15 | 11 and 12 and 13 and 14 | 83 |
| 16 | 6 or 8 or 10 or 15 | 3795 |
| 17 | limit 16 to yr=”2000 - Current” | 2577 |
